# Poorly known 2018 floods in Bosra UNESCO site and Sergiopolis in Syria unveiled from space using Sentinel-1/2 and COSMO-SkyMed

**DOI:** 10.1038/s41598-020-69181-x

**Published:** 2020-07-23

**Authors:** Deodato Tapete, Francesca Cigna

**Affiliations:** Italian Space Agency (ASI), Via del Politecnico snc, 00133 Rome, Italy

**Keywords:** Geophysics, Natural hazards

## Abstract

The instability situation affecting the Middle East poses threats to preservation of cultural heritage. Mapping efforts based on satellite imagery currently concentrate more on recording human-induced damage than impacts of unforeseen natural events (e.g. floods). In 2018, the UNESCO World Heritage Site of Bosra and the landscape of Sergiopolis-Resafa in Syria were flooded after heavy rainfall. While the first incident was reported by heritage organisations (although information was limited to the main monument), the second event was completely unknown. Using optical and radar satellite images from the European Commission’s Copernicus Sentinels fleet and the Italian Space Agency’s COSMO-SkyMed constellation, we prove that these data are an enormous reservoir of information to assess comprehensively the duration, extent and severity of such natural events. In Bosra, several key assets were flooded besides the Roman Theatre, with waters taking from few days to several weeks to evacuate and dry out. In Sergiopolis, while the main ruins were sheltered by the fortification walls, the nearby floodplain was inundated. The floodwater-flow pattern resembled the simulations developed by archaeologists to prove the existence of an ancient system of embankments and dam. Our results suggest that many other events posing risk to heritage assets, otherwise unnoticed, may be unveiled if current satellite imagery archives, yet to analyse, are systematically screened.

## Introduction

Cultural heritage sites are frequently exposed to natural hazards (e.g. floods, earthquakes, landslides)^1^. Research is currently ongoing on how to address the challenges for conservation based on an improved understanding of natural factors determining susceptibility, also in the context of climate change^2,3^. However, the impact of natural hazards is given less attention where more pressing issues arise, such as threats from warfare and looting. This unfortunately applies to various sites across the Middle East and North Africa region, given the widespread situation of political instability. Several initiatives aiming to recording and cataloguing threats include “natural impact” among the damage patterns that are documented using geospatial and satellite data^4,5^. Nevertheless, incident reports and studies mostly focus on anthropogenic hazards. Given the published statistics^5^, we cannot exclude that natural impacts might be under-represented in databases, and more events might have happened, though not documented yet.

Given the constraints due to site inaccessibility in areas under conflict, users exploit satellite data as a reliable source of objective information^6^. To substantiate incident reports collected from broadcast and social media, or written based on direct observation on the ground, bespoke acquisitions at very high spatial resolution have been preferably used^5,7^. However, more studies are increasingly demonstrating the value of high resolution satellite imagery from open access catalogues (e.g. Landsat-8, Copernicus Sentinel-2) for condition assessment^4,8,9^. These image archives are being built in the last 5–7 years through repeated acquisitions collected across the globe, and thus now provide reservoirs of satellite images covering same locations of the Earth’s surface every few days, regardless of specific image orders from users. Such archives become crucial in order to test the above hypothesis that natural hazard events may have occurred, but were not investigated due to lack of reporting or general unawareness.

**Figure 1:**
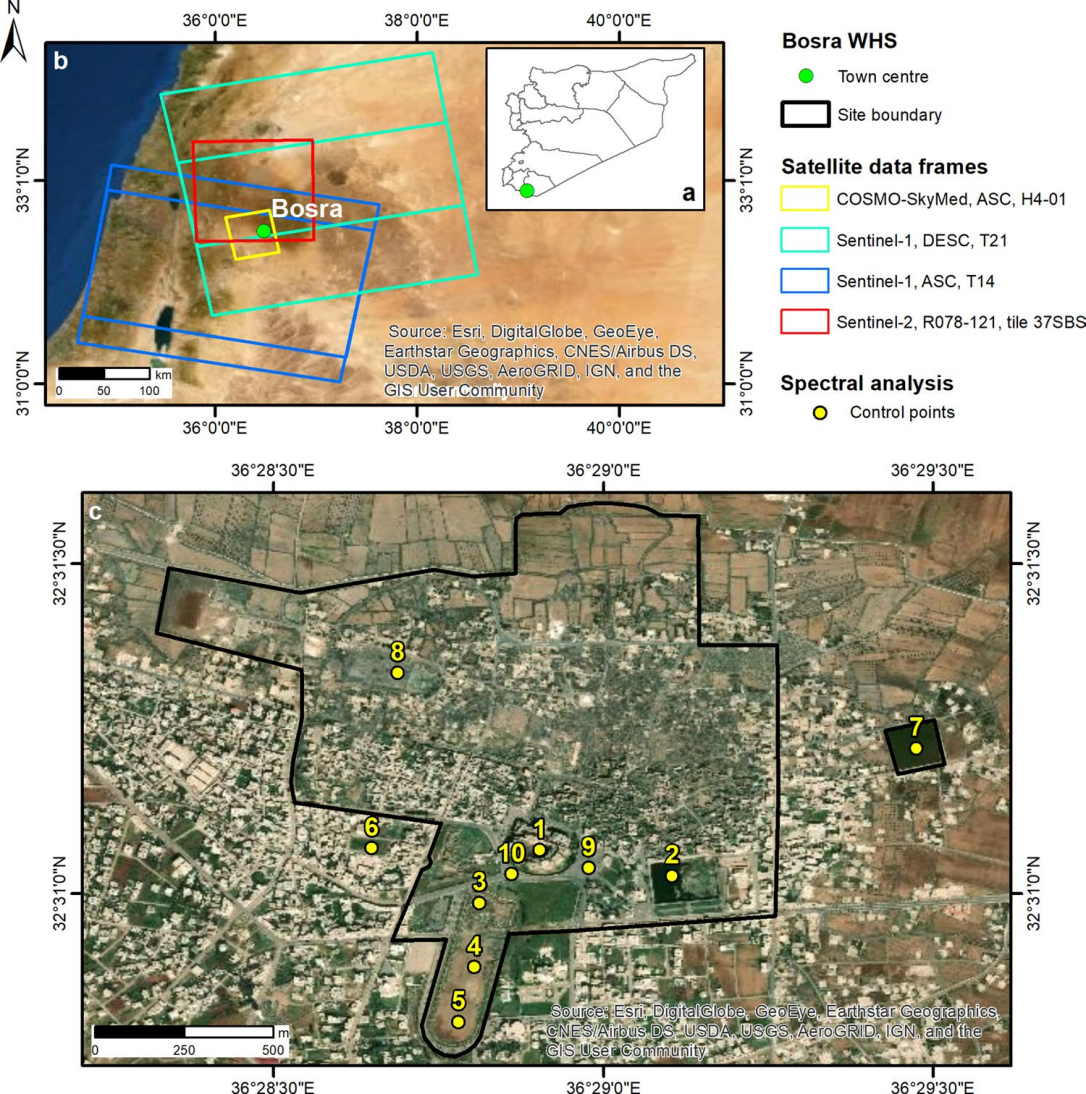
Bosra UNESCO World Heritage Site (WHS). (**a**) Location of Bosra in southern Syria. (**b**) Spatial extent of satellite data analysed to reconstruct the flooding event occurred on 26–29/04/2018. Sentinel-1 and Sentinel-2 footprints retrieved from the Copernicus Open Access Hub. Notation: *ASC* ascending orbit, *DESC* descending orbit, *H* StripMap Himage mode, *T* track, *R* relative orbit. (**c**) Location of control points within the WHS used for spectral analysis of flooding condition at the main monuments and urban sectors: (1) Roman Theatre; (2) Birket al-Hajj (or Pilgrims’ Pool); (3–5) the Field (Hippodrome); (6) lot of bare land; (7) East Pool; (8) water reservoir west; (9–10) roads around the theatre. WHS boundary digitised from Ref.^12^. All maps were generated by the authors using ArcMap v.10.6.1 software (https://desktop.arcgis.com/en/). Background satellite data in (**b**, **c**) are Esri basemaps within ArcMap v.10.6.1 and are credited to: Esri, DigitalGlobe, GeoEye, Earthstar Geographics, CNES/Airbus DS, USDA, USGS, AeroGRID, IGN, and the GIS User Community.

**Figure 2:**
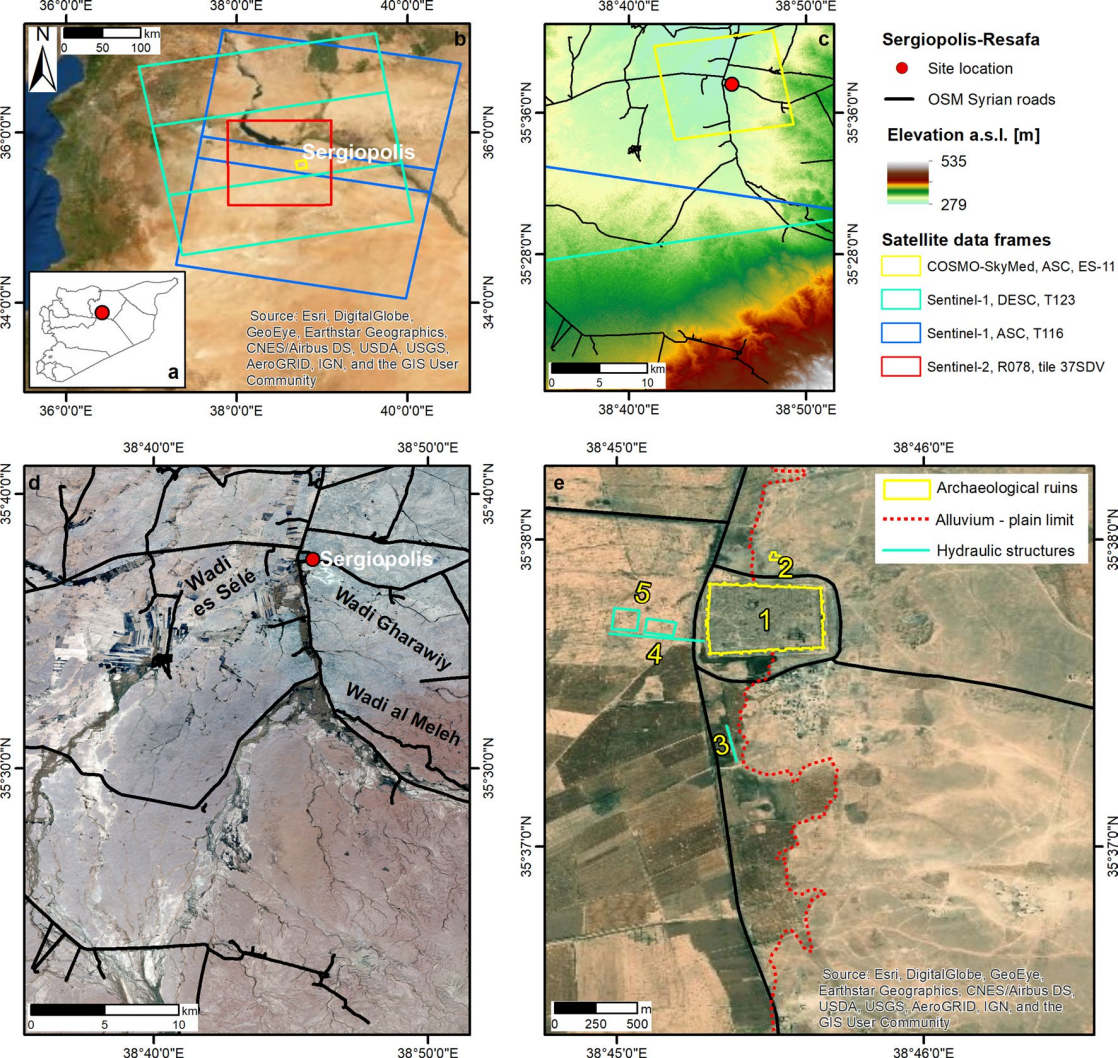
Archaeological site of Sergiopolis-Resafa. (**a**) Location of Sergiopolis in Syria. (**b**) Spatial extent of satellite data analysed to reconstruct the flooding event occurred on 28–30/10/2018. Sentinel-1 and Sentinel-2 footprints retrieved from the Copernicus Open Access Hub. Notation: *ASC* ascending orbit, *DESC* descending orbit, *ES* Enhanced Spotlight mode, *T* track, *R* relative orbit. (**c**) Topography of Resafa Basin based on NASA’s 1 arc-sec SRTM global DEM (30 m ground resolution) distributed by USGS via the LP DAAC Global Data Explorer. (**d**) Situation on 30/10/2018 at the peak of the flooding event. Contains Copernicus Sentinel-2 data 2018. (**e**) Zoomed view of the archaeological site of Sergiopolis prior to the flooding event, with indication of: (1) the fortress enclosing the main buildings, such as the Basilica A; (2) the Ghassanid praetorium; the archaeological finds related to hydraulic structures, i.e. (3) an earthen embankment, (4) an earthen dam and (5) two water basins. Archaeological findings and the alluvium—plain limit are based on maps published in the literature^13,14^. Syrian roads are drawn based on the OpenStreetMap HOTOSM Syrian Arab Republic Roads shapefile accessed through Open Database License at https://data.humdata.org/dataset/hotosm_syr_roads. All maps were generated by the authors using ArcMap v.10.6.1 software (https://desktop.arcgis.com/en/). Background satellite data in (**b**,**e**) are Esri basemaps within ArcMap v.10.6.1 and are credited to: Esri, DigitalGlobe, GeoEye, Earthstar Geographics, CNES/Airbus DS, USDA, USGS, AeroGRID, IGN, and the GIS User Community.

To this purpose, this paper presents the reconstruction of two flooding events that in 2018 hit Bosra UNESCO World Heritage Site (WHS) in the Daraa Governorate and the landscape surrounding the archaeological site of Sergiopolis in the Ar‐Raqqah Governorate, in Syria (Figs. 1, 2). Both sites have unique historical significance and cultural value and, as such, are highly representative of Syrian heritage to protect. Bosra is a major archaeological site in southern Syria (32° 31′ 2.00ʺ N; 36° 28′ 47.00ʺ E; Fig. 1a,b) and is currently inhabited given that over the centuries the namesake modern town has grown up amongst the monuments (Fig. 1c). Owing to the extensive ruins of Nabataean, Roman, Byzantine and Umayyad buildings and the incorporation of the exceptionally intact second century Roman theatre, in 1980 UNESCO recognised Bosra’s high degree of integrity and authenticity, and the WHS was established and became one of the most famous touristic attractions of the country. To the Roman periods also dates back the foundation of Sergiopolis-Resafa, as a fortified settlement of the Eastern Limes, i.e. the Roman border defence system against the Parthian and later Sassanid Empires, 40 km south-west of modern Raqqa (35° 37′ 44.00ʺ N; 38° 45′ 29.00ʺ E; Fig. 2). As the name recalls, the historical significance of Sergiopolis is linked to Saint Sergius, the Roman officer martyred there during the Diocletianic Persecution, to whom the so-called Basilica A was dedicated to mark his grave, thus making Sergiopolis one of the most important Christian pilgrimage sites in the eastern Mediterranean.

Partial to no documentation was available for the two 2018 floods investigated in this paper, respectively. In Bosra, flooding was caused by a severe thunderstorm in late April 2018. Two reports were issued by: (1) the American School of Oriental Research (ASOR) collecting the photographs published in social media^10^; and (2) The Day After Heritage Protection Initiative (TDA)^11^. However, these reports focused on the impact on the Roman Theatre only, and did not include any satellite-based assessment. Therefore, no information was provided to clarify the extent of the event across the WHS, and whether and how the site recovered afterwards. With regard to Sergiopolis, no records of flooding were reported in late 2018 in any of the main information channels or social media, though evidence of floods was found by the authors through a regional screening of satellite image archives.

The two events are here investigated by combining Synthetic Aperture Radar (SAR) and multispectral data collected nearly in the same days by Copernicus Sentinel-1 and Sentinel-2 satellite constellations, respectively, with time series acquired by the Italian Space Agency (ASI)’s COSMO-SkyMed SAR constellation (Figs. 1b, 2b, Tables 1 and 2). The multi-sensor data analysis clarifies the spatial and temporal evolution of the two flooding events, and proves how this combination could bring in new information, otherwise unknown, also with regard to impacts on local heritage assets.

**Table 1:** Satellite SAR data used to analyse the flooding events in Bosra (26–29/04/2018) and Sergiopolis (28–30/10/2018), Syria.

Satellite	Acquisition mode	Date	Geometry	Track/beam	Polarization	Processing level
Sentinel-1	IW	30/04/2018	Ascending	14	VV, VH	GRD
		06/05/2018				
		01/05/2018	Descending	21		
		07/05/2018				
		16/10/2018	Ascending	116		GRD, SLC^a^
		22/10/2018				
		28/10/2018				
		03/11/2018				
		09/11/2018				
		17/10/2018	Descending	123		
		23/10/2018				
		29/10/2018				
		04/11/2018				
COSMO-SkyMed	SM	31/05/2017	Ascending	H4-01	HH	SCS
		11/11/2017				
		01/12/2017				
		21/07/2018				
		25/07/2018				
	SP	31/08/2018	Ascending	ES-11		
		13/09/2018				
		18/10/2018				
		03/11/2018				
		16/11/2018				
		05/12/2018				
		21/12/2018				
		23/02/2019				

Notation: *IW* Interferometric Wide swath, *SM* StripMap Himage, *SP* Enhanced Spotlight, *H* horizontal, *V* vertical, *GRD* level-1 ground range detected, *SCS* level 1A, single-look complex slant products, *SLC* single look complex products.

^a^SLC for the Sentinel-1 image pair (16–28/10/2018) only.

**Table 2:** Sentinel-2 multispectral data used to analyse the flooding events in Bosra (26–29/04/2018) and Sergiopolis (28–30/10/2018), Syria.

Date of acquisition	Cloud coverage	Relative orbit and tile
23/04/2018	Cloud-free	Orbits R078 and R121 Tile 37SBS
26/04/2018	Cloudy and dark	
03/05/2018	Cloud-free	
08/05/2018	Very cloudy	
11/05/2018	Cloud-free	
13/05/2018	Cloudy	
16/05/2018	Cloud-free	
18/05/2018	Cloud-free	
21/05/2018	Cloud-free	
02/06/2018	Cloud-free	
05/10/2018	Cloud-free	Orbit R078 Tile 37SDV
15/10/2018	Hazed	
30/10/2018	Cloud-free	
14/12/2018	Cloud-free	
19/12/2018	Partly cloudy	

Cloud coverage is qualitatively assessed over the study areas only, and not across the whole satellite image frame.

## Results

### Extent and timescale of the 2018 Bosra flood

To establish duration, extent and severity of the 2018 flood at the archaeological site of Bosra in the context of the modern urban layout it is embedded in (Fig. 1a,c), we combined data mining from existing incident reports, with space-based evidence from the Sentinels and COSMO-SkyMed (see “Methods”). Data mining revealed that weather-related threats to conservation of heritage assets at the site were specifically included in the earliest WHS periodic reporting document in 2000^15^, but until April 2018 no report referred to impact of natural hazards. Scholars have concentrated more on anthropogenic hazards arising from looting, bombing, wall collapses, and earth movement^6,16,17^, i.e. those that in 2013 made the site to be included in the List of World Heritage in Danger^18^. The first incident report documenting the flooding event in Bosra WHS is dated 29 April 2018^10^. ASOR collected photographs published by Aleppo Archaeology showing the orchestra of the Roman Theatre flooded up to most of the stage and several rows of seats. Waters also flooded the base of the Ayyubid-era ramparts surrounding the theatre. TDA issued another report on 2 May^11^. Therein, the authors refer that a severe thunderstorm hit Bosra WHS on 26 April and lasted for 3 days. Water quickly accumulated and rose over 3.5 m high, and photographic evidence shows the west and east courtyards flooded up to nearly 1.5 m. Floods also caused sections of the outer walls surrounding the citadel to collapse. This comes out evidently by comparing the photographs published in the ASOR and TDA reports.

No other information was available about this event. It was not mentioned either in the State of Conservation Report issued in February 2019 by the Directorate General of Antiquities and Museums (DGAM)^17^. Therefore, prior to analysing the available archive satellite data, it was not known whether other monuments of the WHS were affected by flooding, and for how long the theatre remained flooded.

The analysis of Sentinel-2 multispectral data clearly testifies that the thunderstorm started on 26 April (see Supplementary). Meteorological data and the CRU TS3.10 Dataset^19^ corroborate this observation, the former via a peak of total daily precipitation recorded on 25–26 April at the King Hussein weather station in Jordan, nearly 27 km south-west of Bosra. Sentinel-2 spectral profiles extracted from control points and polygons located in the main monuments and areas of Bosra WHS (Fig. 1b,c, Table 2), provide a detailed tracking of the whole flooding event, from 23 April up to 2 June 2018.

**Figure 3:**
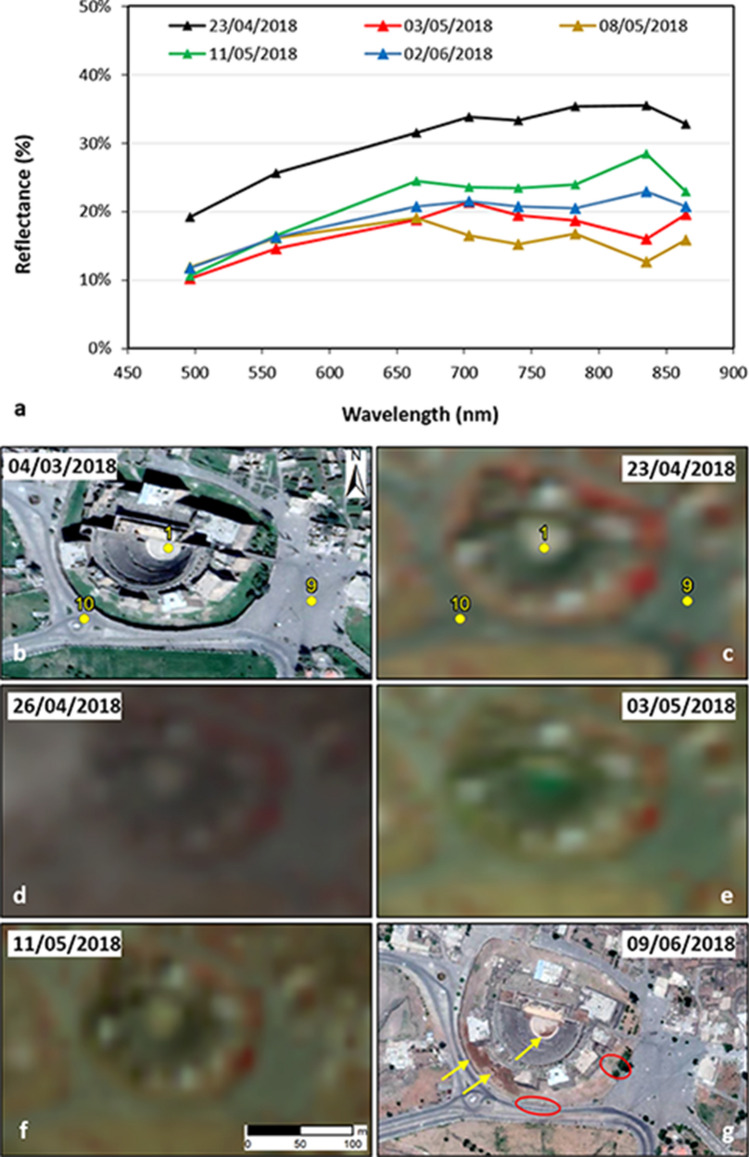
Spectral analysis and visual interpretation of the flooding condition and drying out process in the Roman Theatre of Bosra UNESCO WHS. (**a**) Spectra extracted from Sentinel-2 time series prior to the event (23/04/2018), in the aftermath (03–08/05/2018), and when waters evacuated (11/05–02/06/2018). Temporal evolution of the condition of the Roman Theatre and surrounding roads based on (**b**,**g**) pre- and post-event very high resolution satellite images (less than 1 m) accessed through Google Earth (image credits: 2020 Maxar Technologies), (**c–d**) pre- and (**e–f**) cross-event Sentinel-2 false-coloured IR composites (R: Band 8—NIR; G: Band 4—red; B: Band 3—green; 10 m spatial resolution). Contains Copernicus Sentinel-2 data 2018. Numbered control points in (**b**) and (**c**) as per Fig. 1c. Yellow arrows in (**g**) indicate patches of brown mud partly covering the orchestra, and the darker soil at the base of the western Ayyubid-era ramparts, while red ovals mark the areas of the two collapsed sections of the outer walls surrounding the Ayyubid-era ramparts reported by TDA^11^. Plot in (**a**) was generated by the authors using Microsoft Office Excel 365 (https://www.microsoft.com/en-us/microsoft-365/excel). Maps in (**b**–**g**) were generated by the authors using ArcMap v.10.6.1 software (https://desktop.arcgis.com/en/).

With regard to the Roman Theatre (Fig. 3a), on 3 May the spectral profile confirms the presence of liquid water (i.e. flooding) compared to the pre-event scenario on 23 April. On 8 May the monument was probably still flooded, although it cannot be stated whether at the same depth as on 3 May. In the following days, waters started progressively evacuating from the orchestra and the stage, as revealed by the shape of the spectral profiles that turn back to those prior to the event (i.e. on 23 April), though with lower reflectance (Fig. 3a). Spectra differ at Bands 5 to 7 (from 704 to 783 nm), probably due to the presence of mud left by waters.

**Figure 4:**
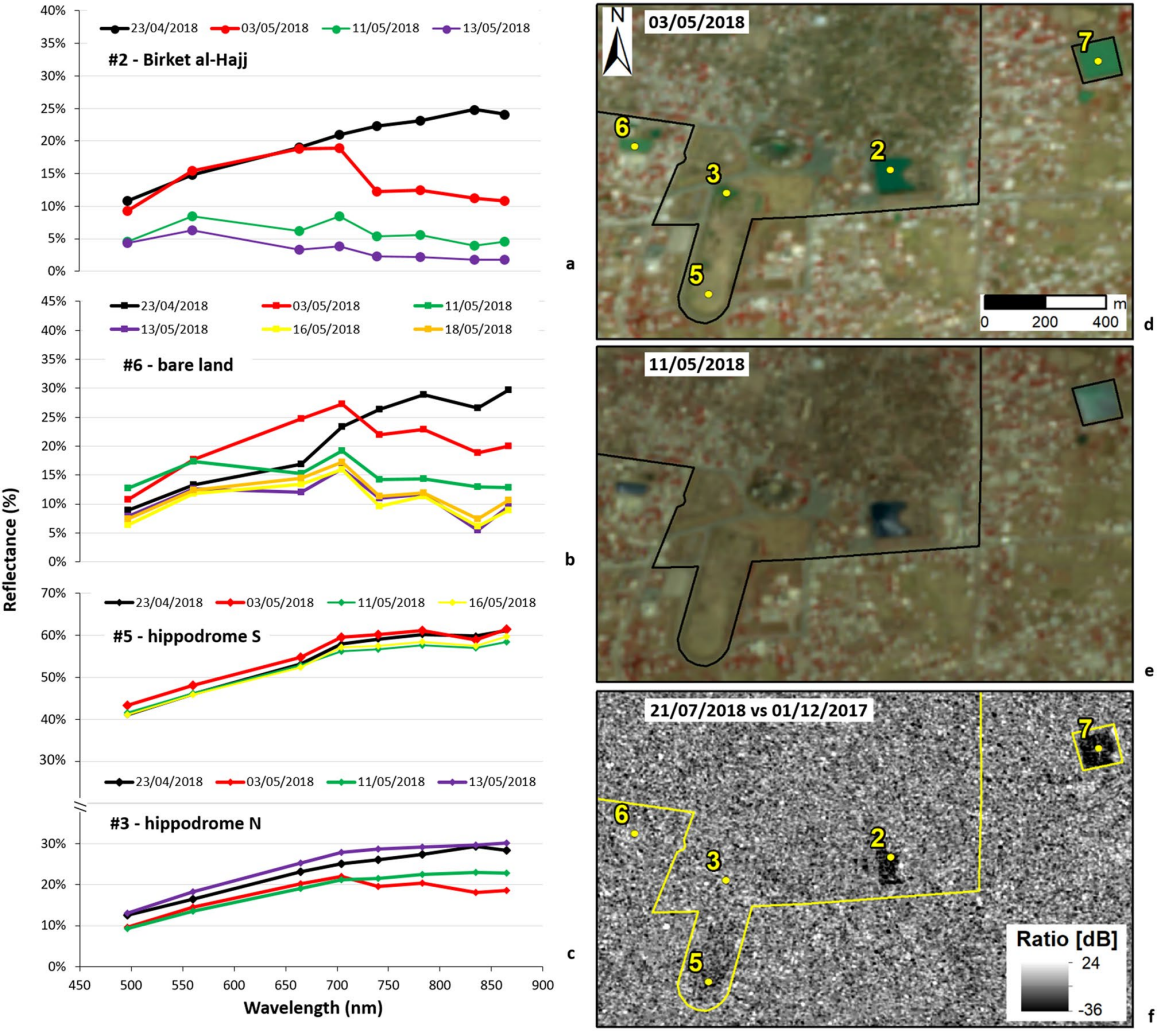
Spectral analysis of the flooding condition and drying out process in: (**a**) Birket al-Hajj (or Pilgrims’ Pool); (**b**) lot of bare land; (**c**) the Field (Hippodrome). Visual interpretation of site condition based on Sentinel-2 false-coloured IR composites (R: Band 8—NIR; G: Band 4—red; B: Band 3—green; 10 m spatial resolution) collected (**d**) in the aftermath (03/05/2018) and (**e**) a few days after the peak of the flooding event (11/05/2018). Contains Copernicus Sentinel-2 data 2018. Numbered control points in (**a**) and (**c**) as per Fig. 1c. (**f**) Cross-event SAR amplitude ratio map from COSMO-SkyMed StripMap Himage (3 m ground resolution) scenes acquired in ascending mode on 01/12/2017 and 21/07/2018 (COSMO-SkyMed Products credits: ASI—Italian Space Agency—2017–2018. All Rights Reserved). WHS boundary digitised from Ref.^12^. Plots in (**a**–**c**) were generated by the authors using Microsoft Office Excel 365 (https://www.microsoft.com/en-us/microsoft-365/excel). Maps in (**d**–**f**) were generated by the authors using ArcMap v.10.6.1 software (https://desktop.arcgis.com/en/).

The multi-temporal spectral analysis over the Birket al-Hajj and the East Pool shows that both remained flooded under deep waters across the whole investigated period, as clearly highlighted by sun-glint-free spectra (Fig. 4a). Same is found for the 100 m by 50 m bare land lot, located west of the Necropolis of Tell Aswad and the Odeon (Fig. 4b). On the contrary, control points in the central and southern parts of the hippodrome were almost dry and never flooded (Fig. 4c). Whereas, the northern part, initially covered by surface liquid water on 3 May, was mostly dried out on 11 May (Fig. 4c). Two days after, on 13 May, the spectral profile matched the pre-event situation.

Driven by the spectral analysis, visual inspection of Sentinel-2 images was straightforwardly focused on those areas of the WHS that were flooded. This examination confirms the above conclusions (Fig. 3c–f, Roman Theatre; Fig. 4d,e, reservoirs, bare land and hippodrome). A wider view of the countryside around Bosra also highlights that, outside the WHS, flooding sparsely affected fields and watercourses across the landscape, but no wide areas were inundated.

Unfortunately, the Sentinel-1 SAR images (see Table 1), did not allow us to better refine the multi-temporal analysis. This was due to the coarse Sentinel-1 spatial resolution and, more importantly, unforeseen signal interference affecting the SAR images.

More informative was, instead, the COSMO-SkyMed time series. The cross- and post-event images covering up to 25 July confirm that the East Pool and Birket al-Hajj were still flooded, with the eastern half of the latter already dried out (Fig. 4f). At that time, the bare land was completely dry and free from waters. Additionally, the cross- and post-event ratio maps do not highlight change patterns due to permanent structural damages (Fig. 4f). However, limitations due to the spatial resolution and visibility along the satellite line-of-sight could have hampered the possibility to capture the outer wall collapse reported by TDA^11^.

### Discovery of the 2018 Sergiopolis flood

To investigate the potential occurrence of flooding events at Sergiopolis (Fig. 2a,b), we combined the analysis of the site conservation literature with thorough regional screening of satellite image archives, by exploiting change detection approaches based on moisture and flood indexes from multispectral imagery, as well as SAR amplitude changes and coherence (see “Methods”). In the heritage conservation literature, Sergiopolis has been repeatedly reported for being affected by looting (mostly occurred prior to the recent conflict^20,21^ and distinctively visible in satellite imagery^9^), theft, vandalism^22^ and militarization^23^ (in more recent times) that mostly affected the archaeological monuments within the massive walls of the fortress (#1 in Fig. 2e). Neither the reports mention natural hazard events occurred at the site in the last years, nor Sergiopolis was referred to in the news published online about the flash floods affecting various areas across the Middle East between 20 and 31 October 2018^24^.

Conversely, our regional screening of Sentinel-2 archives allowed the discovery of an unknown event affecting the site during that month. The comparison between the two closest cloud-free Sentinel-2 images collected on 5 and 30 October (Fig. 2d) highlighted an unequivocal flooding pattern across the Resafa hydrological basin which intersected the location of the ruins. This evidence suggested the opportunity to investigate more in detail the spatial and temporal evolution of the event.

**Figure 5:**
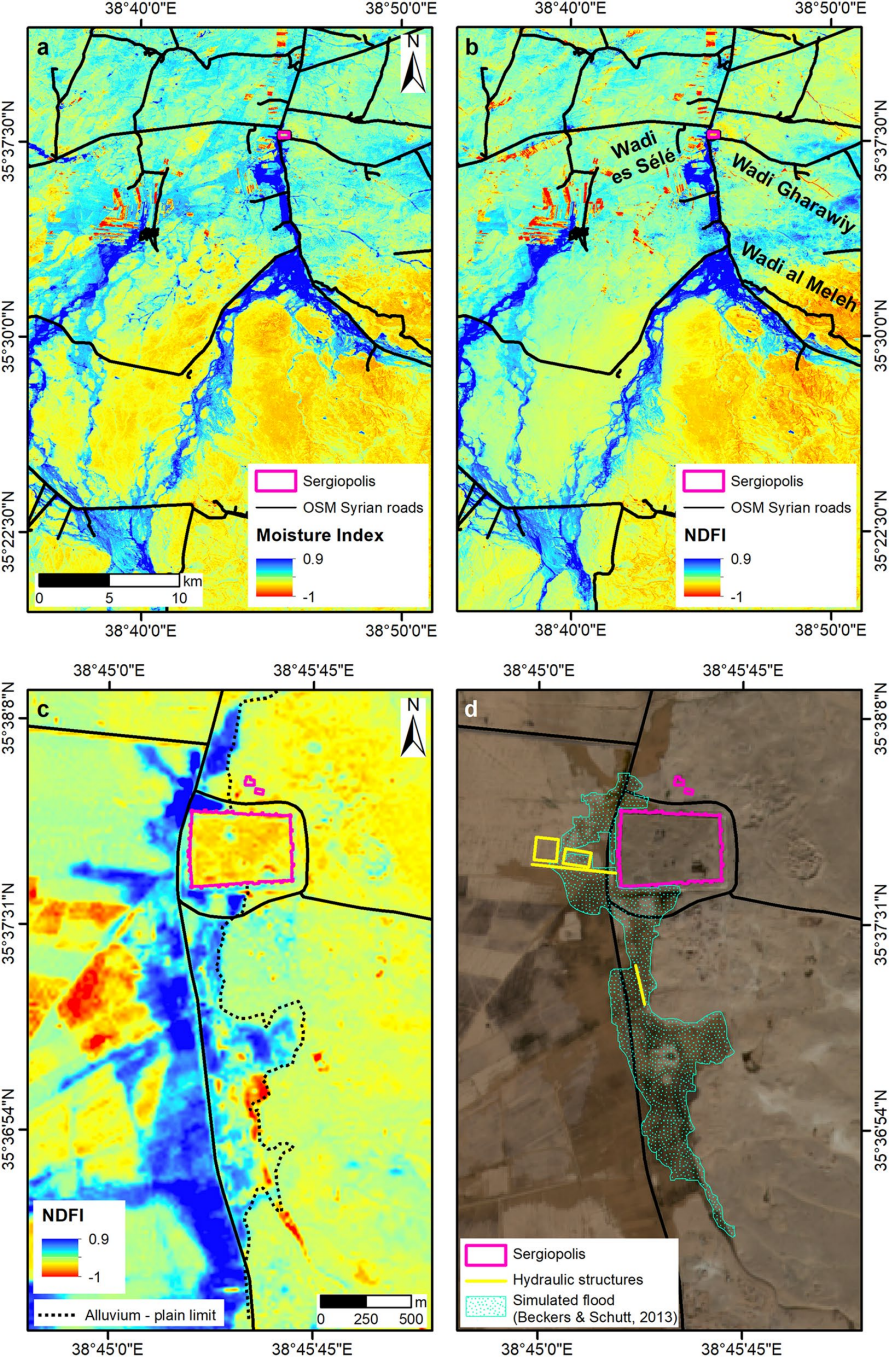
Spatial extent of floods in Resafa Basin and Sergiopolis as highlighted by means of (**a**) Moisture Index and (**b**) Normalized Difference Flood Index (NDFI) maps (20 m spatial resolution) generated from the Sentinel-2 image collected on 30/10/2018. Zoomed view of Sergiopolis to compare (**c**) the NDFI map and (**d**) the original true-colour image (R: Band 4—red; G: Band 3—green; B: Band 2—blue; 10 m spatial resolution). Contains Copernicus Sentinel-2 data 2018. Archaeological findings, the alluvium—plain limit and the extent of the simulated flood are based on maps published in Refs.^13,14^. Syrian roads are drawn based on the OpenStreetMap HOTOSM Syrian Arab Republic Roads shapefile accessed through Open Database License at https://data.humdata.org/dataset/hotosm_syr_roads. All maps were generated by the authors using ArcMap v.10.6.1 software (https://desktop.arcgis.com/en/).

The Normalized Difference Flood Index (NDFI) map of the 30 October Sentinel-2 image delineates very well the spatial extent of the flooded areas (Fig. 5b,c). A similar result is obtained with the Moisture Index map (Fig. 5a), although other wetness patterns are observed due to soil moisture. The nearby of Sergiopolis was flooded as a consequence of the inundation coming from the upper part of the Resafa basin, and particularly from the Wadi al Meleh and its left bank tributaries (Fig. 5a,b). As visible in the SRTM DEM (Fig. 2c), Sergiopolis is situated in the Syrian desert steppe at the eastern bank of the Wadi es Sélé (Fig. 2d), downstream of the confluence of several major tributaries, at an altitude ranging from 284 to 303 m a.s.l. Therefore, the topography is a key predisposing factor for flooding. Furthermore, the October 2018 event was of wider regional character. The NDFI map shows inundation of similar severity to the west, with waters overrunning some agricultural fields (Fig. 5a,b).

**Figure 6:**
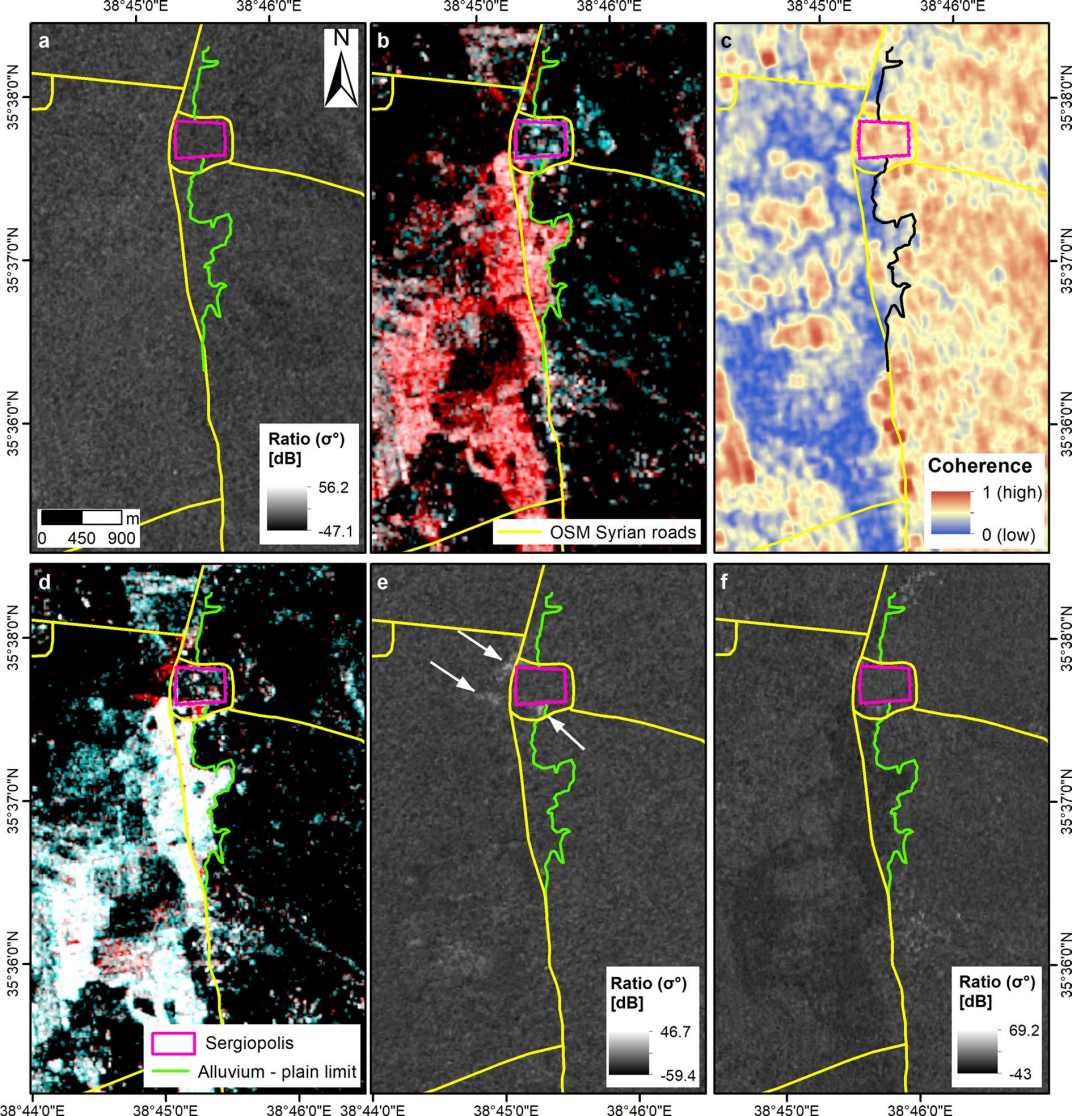
Multi-temporal analysis of the flooding event in Sergiopolis through the Sentinel-1 Interferometric Wide swath (spatial resolution 20 m in azimuth by 5 m in range) time series. (**a**) SAR amplitude ratio map from the pre-event pair acquired in ascending orbit, VV polarization, on 16/10/2018 and 22/10/2018. (**b**) RGB colour composite of the cross-event pair, ascending, VV, R: 28/10/2018, G and B: 16/10/2018 and (**c**) the matching interferometric coherence map. (**d**) RGB colour composite of the cross-event pair, ascending, VV, R: 03/11/2018, G and B: 28/10/2018 and (**e**) the matching ratio map. White arrows indicate patterns of increased radar backscatter in the nearby of Sergiopolis fortification walls and water basins. (**f**) Ratio map of the post-event pair, descending, VV, 04/11/2018 and 29/10/2018. Contains Copernicus Sentinel-1 data 2018. Archaeological findings and the alluvium—plain limit are based on maps published in Refs.^13,14^. Syrian roads are drawn based on the OpenStreetMap HOTOSM Syrian Arab Republic Roads shapefile accessed through Open Database License at https://data.humdata.org/dataset/hotosm_syr_roads. All maps were generated by the authors using ArcMap v.10.6.1 software (https://desktop.arcgis.com/en/).

No other Sentinel-2 image provides a clear sky view of Sergiopolis just before or immediately after the event. The 15 October scene is hazed (although the visibility is sufficient to infer that the area was not yet flooded at that time) and the next cloud-free image was collected on 14 December 2018, when the event was already over. The event can be further constrained temporally by using the Sentinel-1 time series, in both ascending and descending orbits and Vertical–Vertical (VV) and Vertical-Horizontal (VH) polarizations. The Sentinel-1 ratio maps 23 vs. 17 October (descending, VV) and 22 vs. 16 October (ascending, VH and VV; Fig. 6a) do not show any signs of flooding. Instead, the ascending Sentinel-1 image collected on 28 October pre-dates the start of the flooding of at least 2 days, compared to the Sentinel-2 data stack. The red pattern in the RGB colour composite (Fig. 6b) and the matching blue-coloured pattern in the coherence map (Fig. 6c) mark the extent of the flooded landscape south and in the nearby of Sergiopolis. At larger scale, swollen rivers and flooded land appear as brighter areas of increased radar backscatter in the VV polarized image better than in the VH one. This would suggest that waters were generally not deep and the terrain soon became wet and muddy, thus matching the brownish tone visible in the 10 m RGB composite of the 30 October Sentinel-2 image (Fig. 5d).

On 3 November the situation was likely more or less the same across the basin. The upper part of the basin was still flooded on 3 and 4 November, and the RGB composite and the ratio map (3 November vs. 28 October, ascending, VV) do not reveal evident change (Fig. 6d,e). The only exception is represented by some patterns of increased radar backscatter localised south of the fortification walls, in front of their NW corner and south of the water basins (see white arrows in Fig. 6e).

**Figure 7:**
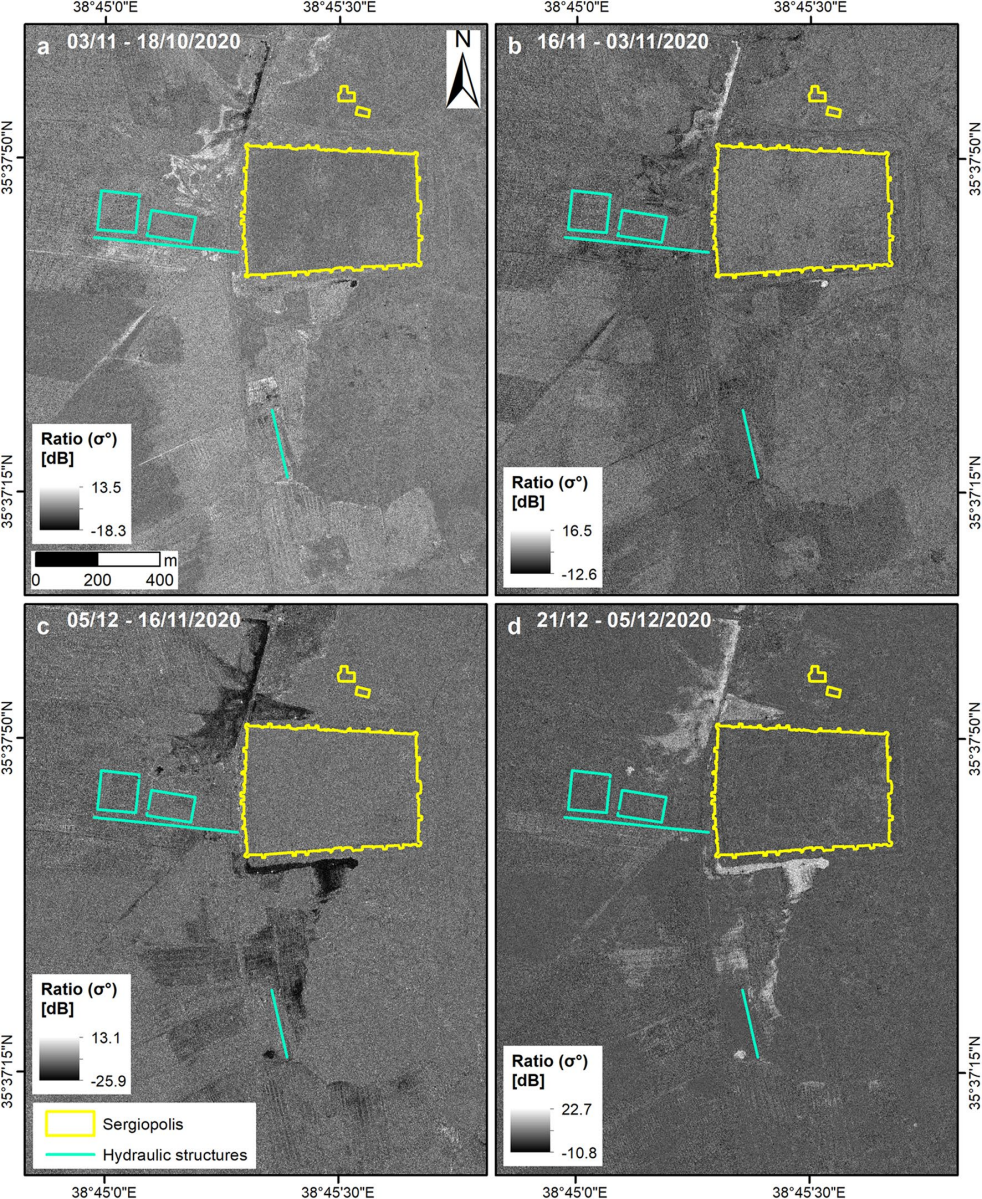
Multi-temporal analysis of the flooding event in Sergiopolis through the COSMO-SkyMed Enhanced Spotlight (~ 1 m ground resolution), ascending orbit, HH polarization, SAR amplitude ratio maps generated from: (**a**) the cross-event pairs 18/10–03/11/2018 and (**b**) 03–16/11/2018; and the post-event pairs (**c**) 16/11–05/12/2018 and (**d**) 05–21/12/2018 (COSMO-SkyMed Products credits: ASI—Italian Space Agency—2018. All Rights Reserved). Archaeological findings are based on maps published in Refs.^13,14^. All maps were generated by the authors using ArcMap v.10.6.1 software (https://desktop.arcgis.com/en/).

These patterns are very finely captured by the cross-event COSMO-SkyMed SAR Enhanced Spotlight image collected at 1 m spatial resolution on 3 November (i.e. the same day as the Sentinel-1), and its ratio with the pre-event 18 October image (Fig. 7a). In front of the NW corner of the fortress, the mixed radar backscatter ratio patterns are compatible with land partly covered by nearly flat backwater (dark areas) and partly by muddy waters (brighter areas). On the contrary, the basins of the ancient water harvesting system of Sergiopolis (#5 in Fig. 2e and Fig. 5d) were dry (Fig. 7a), thus confirming the Sentinel-2 observations on 30 October (Fig. 5c,d).

Totally reverse patterns are, instead, found in the 16 November COSMO-SkyMed image (Fig. 7b), so by then, presumably, waters should have already evacuated and the soil dried out. A confirmation, in this regard, is also found in the Sentinel-1 ratio between 4 November and 29 October VV polarized scenes (Fig. 6f), where a clear decrease in the radar backscatter is observed along the road leading to Sergiopolis and in the fields to the west (to compare also with Fig. 6b,c). Curiously, on 5 December, when it is plausible that the flooding event was over, the COSMO-SkyMed image shows wide marked patterns of decreased backscatter along the southern walls of Sergiopolis, along the road opposite to the NW corner of the fortress and in some of the agricultural fields south of the site (Fig. 7c). In absence of coeval multispectral image, it is difficult to interpret unequivocally what these patterns in the ratio map were due to, though the marked decrease in the radar backscatter suggests that they could be related to surface water. However, on 21 December they are replaced by reverse patterns and definitely no signs of flooding are visible anymore (Fig. 7d). This matches with the evidence in the Sentinel-2 images collected on 14 and 19 December, which show that inside Sergiopolis and in some of the fields the soil was greening.

## Discussion: the value of digging into satellite archives

With regard to the Bosra flood, satellite data corroborated most of the information available in the few published reports and, more importantly, expanded our knowledge about the spatial distribution and temporal evolution of the event. It is confirmed that the intense rain causing the flood started on 26 April 2018, and that the orchestra and stage of the Roman Theatre were flooded soon after. In absence of other on-the-ground evidence, satellite data were essential to ascertain that other monuments were flooded, i.e. the Birket al-Hajj, the East Pool, as well as the northern part and small areas in the centre of the hippodrome. This information could not have been known otherwise. Given their focus on the key archaeological landmark, the published reports could not be used alone to retrieve a full condition assessment of the whole WHS.

The site recovered relatively quickly from the event. Waters gradually left the Roman Theatre in the following days, with at least the orchestra and stage evacuated by 11 May, when it appears that no water but mud was still present (Fig. 3f). At the same time, the most affected portion of the hippodrome was already dry (Fig. 4e), whereas, Birket al-Hajj and the East Pool were still under deep waters until July (Fig. 4f). At that time, the other most evident area testifying that the town was hit by flooding (i.e. the bare land lot) was completely dried out (Fig. 4f).

From a technical point of view, the identification of the flooded areas and tracking of the temporal evolution were made agile owing to the semi-automatic spectral analysis of the check points. This approach overcame the limitation of the visual inspection of the satellite time series, which was later carried out for validation more expeditiously. Despite the limitations in the available datasets (i.e. signal interference affecting Sentinel-1 data; lack of COSMO-SkyMed images during the event), the multi-sensor integration was effective to achieve an overall understanding of the event, and proved the complementarity between Copernicus and Contributing Missions data.

The examination of the Google Earth time lapse (i.e. one of the open tools mostly used by heritage science researchers and practitioners^4,5^) suggests that in this case same (or better) reconstruction could not have been made using very high resolution (i.e. less than 1 m) satellite imagery. Despite the availability of a pre-event WorldView-2 (4 March 2018, 0.59 m ground sample distance—GSD; Fig. 3b) and a post-event WorldView-3 image (9 June 2018, 0.35 m GSD; Fig. 3g), the temporal interval of this image pair is much longer than the overall timeframe of the flooding event. Suspicious signs that could have been observed would have been the brown mud partly covering the orchestra and the darker soil at the base of the western Ayyubid-era ramparts (see yellow arrows in Fig. 3g). However, with no prior knowledge that a flooding event occurred at the site, these features may not have been necessarily attributed to flooding. No more informative could have been the fact that the Birket al-Hajj and the East Pool appear under deep waters. In absence of images collected few days apart, this is not an exceptional feature per se. These reservoirs are regularly filled in by waters. The only anomalous element compared to the pre-event situation is, instead, the waterlogged bare land visible on the 9 June image, but this evidence alone is far too limited to understand the overall impact of the 2018 flood across the WHS.

On the contrary, guided by the pictures reported by TDA^11^, in the post-event image it is possible to identify the two collapsed sections of the outer walls surrounding the Ayyubid-era ramparts (see red polygons in Fig. 3g). Although the observation is slightly hampered by the illumination and feature visibility, the higher resolution of the WorldView-3 image proved beneficial to fill the gap in the analysis based on Sentinel-1, Sentinel-2 and COSMO-SkyMed data. Such comparison with Google Earth would not have been possible in the case of Sergiopolis, given that the only pre-event image was collected on 5 April 2018 and no post-event scene since then is available. This lack of satellite imagery also reflects the gap in the archives of very high resolution satellites (e.g. Maxar DigitalGlobe fleet). Therefore, the Sentinel-1/2 and COSMO-SkyMed data analysed in this paper are essential to record this event that, otherwise, would not have been known.

The relevance and implications of such digital documentation to the conservation of Sergiopolis come out more clearly when the 2018 flood is contextualised with regard to the topographical, environmental and archaeological setting of the site. As mentioned above, the downstream location within the river basin is a predisposing factor (Fig. 2c, d), with the western part of the town lying in the floodplain and most of the ruins on the elevated plain extending to the east^13,14^ (see the alluvium—plain limit in Fig. 2e). Therefore, the topographic position definitely favours the accumulation of excess water during heavy rainfall. This natural process is believed to have been intentionally exploited in ancient times by local inhabitants to counterbalance the absence of perennial water sources. Two water-harvesting techniques were exploited^14^. First, the collection and storage of rainfall and runoff waters in bottle-shaped cisterns (some are still visible inside the walls and others were found in the Wadi es Sélé) were one of the ways to supply the town with drinking water. Second, a floodwater-harvesting system, consisting of a series of embankments (of which a north–south running section, nearly parallel to the modern road, is attested in the survived archaeological findings; structure #3 in Fig. 2e) facilitating the floodwater harvesting up to an earthen dam (#4), located south of the water basins built west of the fortress walls (#5 in Fig. 2e)^25^. The reliability of such system was recently proved by means of hydraulic modelling assuming the existence of the enbankments^13,14^. This system should have worked well because of the geographic position of Sergiopolis, close to the 200 m isohyet, at the margin of a climate region characterised by precipitation regime with strong Mediterranean character (i.e. regular rainfall events and low variability)^13^.

No daily precipitation data to constrain the October 2018 flood were found. On the other side, the CRU TS3.10 Dataset for the years 2017 and 2018^19^ agrees with the climate diagram of Resafa (1983–2011)^13^, and confirm that October is the month when precipitations start again after four month-long (June–September) hot and dry season. October 2018 was not an exception and the trend was increasing in the following November–December. Therefore, it is not surprising that the regional character flooding event affecting Sergiopolis occurred at the end of October.

As discussed above, Sentinel-2 NDFI and Moisture Index (Fig. 5a–c), the Sentinel-1 ratio maps and RGB composites (Fig. 6), and the COSMO-SkyMed products (Fig. 7) mutually complement each other in delineating the flooded areas at the peak of the event.

If these maps are compared with the results of the flow pattern analysis^13^ (Fig. 5d), a partial match can be noted between the extent of the areas flooded in October 2018 and the scenario identified with the simulations. Nevertheless, some differences exist. During the event, the majority of the water flow came from the Wadi al Meleh and tributaries, and not from the Wadi Gharawiy as per the simulation (Fig. 5a–c). Therefore, the lateral spreading of the inundation was much wider towards the west, with waters expanding across both sides of the modern road (Fig. 5c, d). As per the hydraulic model (Fig. 5d), the main flow reached the valley embayment to the east and inundated the small nearly-squared plain located east (Figs. 5c, 6b, c and 7a). Waters then flowed to the north, reaching the southern walls of the town. There, they accumulated and the flood was likely retained south of the two water basins. Differently from the model, the excess water was not channelled through the spillway between the water basins, but flowed following the modern road, to reach the depression north of Sergiopolis. Flooded areas are found about 600 m further north, but they did not include the Ghassanid praetorium, owing to its higher topographic position.

It can be concluded that the 2018 event somehow validates the model, and demonstrates that flooding is a likely natural hazard in Sergiopolis. Assuming that the archaeological hypothesis about the floodwater-harvesting system is correct, and that this system was used in the past to transform a threat into a resource for the town, the current situation by which this system is not functioning anymore and no maintenance or management measures are undertaken at the site, leaves Sergiopolis much more exposed to flooding events than in ancient times. From a technical point of view, the multi-sensor integration achieved in Sergiopolis not only strengthens the case to investigate the available satellite image archives and to combine SAR and optical observations, but also proves the benefit of creating bespoke and/or background acquisitions at high spatial and temporal resolution over hotspot areas and key heritage assets (such as those tasked with COSMO-SkyMed over Sergiopolis). While in this research we focused on Copernicus Sentinels and SAR Contributing Missions, other very high resolution satellite imagery, e.g. PlanetScope, could be encompassed as part of the methodological approach, in order to increase the observation capabilities, and move towards a more synergistic and coordinated use of satellite sensors and their image archives (as per the novel concept of “Virtual Constellations”^26^). Such increased accessibility to imagery (and processing tools) should also support more widely the applicability of the approach presented in this paper and its exportability. Provided that satellite imagery is available to cover past events, the research workflow also relies on ancillary information such as meteorological records and hazard event inventories, to be browsed to find a temporal match with the evidence extracted from the satellite images or, vice versa, to identify which geographic area to look at and, the temporal period when an event could have occurred.

It is very likely that many other natural hazard events than the two analysed in this paper occurred across heritage sites in the Middle East and North Africa region, and they are still from poorly documented to completely unknown, with limited to no on-the-ground evidence available. Therefore, this research is novel in that it demonstrates the feasibility, alongside limitations, of digging into the satellite archive data to enrich existing database records, and thus fill the knowledge gap about natural processes of potential concern for site conservation, in addition to anthropogenic processes that are currently much more documented. To this purpose, as proved by recent and current initiatives^4,5^, such a systematic screening of satellite archives could contribute to inform mitigation measures if a workflow was established to share the information gained from these geospatial datasets with local stakeholders and authorities who are in charge of heritage protection and preservation. While the results presented in this paper are outcomes of scientific research, they prove that, in case such integrated satellite observations were embedded in an ad hoc workflow, they could provide the necessary evidence base for mitigation measures, by: sensitizing stakeholders about hazards that are given less attention but anyway require to be accounted for over a longer timescale; showing the impact that unknown (or poorly documented) events have had on local heritage and landscape; and consequently allowing the projection of the impacts that future events of similar severity could cause if no actions were put in place.

## Methods

### Satellite data

The selection of input data used in this work aimed to demonstrate the complementarity between the Sentinels and COSMO-SkyMed, according to the concept of the Copernicus Earth Observation and Monitoring programme^27^ and its Contributing Missions^28^. Tables 1–2 list the satellite SAR and multispectral data used to analyse the two flooding events. Data frames and respective spatial coverage over Bosra and Sergiopolis are shown in Figs. 1b and 2b.

Sentinel-1 data are SAR images acquired by the namesake constellation of the Copernicus Programme in Interferometric Wide swath mode, with spatial resolution of 20 m in azimuth by 5 m in range^29^. We mainly used Level-1 Ground Range Detected (GRD) products, i.e. focused SAR data that were detected, multi-looked and projected to ground range using an Earth ellipsoid model, and thus contain amplitude information only. Sentinel-1 are increasingly being used for condition assessment of cultural landscapes^30^.

Sentinel-2 data are acquired every 5 days by the Multispectral Instrument (MSI) on-board the two twin satellites of the Copernicus Sentinel-2 constellation, in 13 bands spanning from 443 to 2,202 nm (i.e. visible—VIS, Near InfraRed—NIR, Short-Wave InfraRed—SWIR)^31^. We used Bottom of Atmosphere (BOA) reflectance in cartographic geometry, i.e. Level 2A products based on automated processing of Top-Of-Atmosphere (TOA) reflectance in cartographic geometry (i.e. Level 1C) products with the Sen2Cor processor and PlanetDEM Digital Elevation Model^32^. Images listed in Table 2 were selected from the available archive, by removing those totally hampered by clouds over the study area. Sentinel-2 has been proved suitable for systematic condition assessment of heritage assets^8^.

COSMO-SkyMed data are collected from the COnstellation of small Satellites for Mediterranean basin Observation (COSMO-SkyMed) mission of the Italian Space Agency (ASI)^33,34^, made of four identical space-crafts (i.e. CSK1, CSK2, CSK3, and CKS4), each equipped with multimode SAR sensor operating in the X-band (9.6 GHz frequency; 3.1 cm wavelength).

The dataset covering Bosra UNESCO WHS (Table 1; Fig. 1b) is a selection of the time series being collected in StripMap Himage mode (3 m spatial resolution) along ascending orbits as part of the COSMO-SkyMed Background Mission, i.e. a low priority acquisition plan covering large and capital cities, populated areas, sensible areas for geohazards and over 260 UNESCO WHS on a regular basis^35^. Three pre-event and two post-event scenes were selected from the available catalogue. The selection was constrained by the acquisition gap of nearly six months from December 2017 to mid-July 2018.

The dataset covering Sergiopolis (Table 1; Fig. 2b) is instead a selection of the tailored time series tasked in Enhanced Spotlight mode (1 m spatial resolution), in HH polarization, ascending orbit, since summer 2018.

These data were accessed through a license to use by ASI. One of the existing mechanisms for scholars and users to access COSMO-SkyMed data to undertake similar research is ASI’s “Open Call for Science”^36^. While restrictions and limitations may apply (e.g. in terms of geographical areas, total number of images that can be requested), the call is open to international scientific investigators and offers the opportunity to access archive imagery (including those already covering heritage sites^35^), as well as to task a limited quota of new acquisitions.

### Flooding condition assessment through Sentinel-2 spectral analysis

In the case of Bosra WHS, one of the main aims of the multi-temporal analysis was to assess which monuments and archaeological sectors of the site were flooded and, if so, for how long. This could have been simply done by visual inspection of Sentinel-2 images. However, such an approach could be time-consuming if several points need to be checked, is subjective and dependent on the operator’s expertise and site knowledge. Therefore, control points and polygons were located in the key areas of WHS (Fig. 1c): inside the stage and orchestra floor of the Roman Theatre; in the roads nearby the Roman Theatre; along the longitudinal axis of the hippodrome; the Birket al-Hajj or Pilgrims’ Pool in the south-east; the East Pool; the water reservoir west^12^. Additionally, some points and areas were located in bare land sectors that could have been flooded given their land use and topography. To achieve an accurate positioning, this was done by using the pre-event WorldView-2 image collected on 4 March 2018 and available in Google Earth. Zonal statistics were then extracted for the control points and areas to derive the spectral profiles from Band 2 (Blue, 492 nm) to Band 8A (Vegetation Red Edge, 865 nm). At each date of acquisition, the spectral profile was classified as either “flooded” or “dry”, according to reference reflectance spectra of water, bare soil and artificial surfaces from spectral libraries. The multi-temporal comparison of the spectral profiles highlights when and where a monument or archaeological sector of Bosra WHS was flooded, for how long it remained flooded, and the time-scale during which it progressively dried out.

### Identification of flooded areas through Sentinel-2 moisture and flood indexes

In the case of Sergiopolis, the Moisture Index and the Normalized Difference Flood Index (NDFI) were calculated in ArcMap v.10.6.1 as follows:

$$Moisture Index = \frac{{\left( {Band 8A - Band 11} \right)}}{{\left( {Band 8A + Band 11} \right)}}$$

where, for Sentinel-2, Band 8A is the Vegetation Red Edge centred at 865 nm, and Band 11 is the SWIR band at 1614, both acquired at 20 m spatial resolution, thus resulting in a raster of moisture index map of equivalent spatial resolution.

$$NDFI = \frac{{\left( {Band 4 - Band 12} \right)}}{{\left( {Band 4 + Band 12} \right)}}$$

according to the definition by Boschetti et al.^37^, where, for Sentinel-2, Band 4 is the Red band centred at 665 nm, and Band 12 is the SWIR band at 2,202 nm. The NDFI raster of each Sentinel-2 image was obtained by using the original 20 m Band 12 and the 20 m resampled Band 4 image layers accessed through the Copernicus Open Access Hub.

### Identification of flooded and drying out areas through SAR amplitude change

Sentinel-1 images were processed to extract patterns of radar backscatter change due to flooding and drying out in the cross- and post-event SAR image pairs. The processing was performed using the SNAC—SNAP S-1 GRD Amplitude Change processing tool^38^ developed by the European Space Agency (ESA) and available in ESA’s Geohazards Exploitation Platform (GEP)^39^. The SNAC tool ingests pairs of Sentinel-1 images in GRD format and, after co-registration, calibration and terrain correction, generates calibrated and terrain corrected [decibel, dB] slave and master backscatter products (i.e. post- and pre-event data), and RGB composites based on backscatter values in dB.

The slave and master products were then imported in ArcMap v.10.6.1 and used to generate the amplitude change detection map through ratioing^40^:

$$R_{{\frac{{t_{s} }}{{t_{m} }}}} \left( i \right) = \frac{{\sigma_{{t_{s} }}^{0} \left( i \right)}}{{\sigma_{{t_{m} }}^{0} \left( i \right)}}$$

where *t*_*s*_ and *t*_*m*_ are the acquisition times of the slave and master products respectively, *σ*^0^ is the normalised radar backscatter (linear scale). Change detection products were generated for both polarizations, i.e. the “like-polarization” VV and the “cross-polarization” VH.

Furthermore, the ascending SAR image pair (16–28/10/2018) was also processed with GEP’s COIN—Coherence and Intensity change for Sentinel-1 service^41^. In this case, Sentinel-1 images were processed starting from Single Look Complex (SLC) format to generate the interferometric coherence map, the backscatter intensity difference in dB, as well as the RGB composite (red channel: *σ*^0^ slave; green: *σ*^0^ master; blue: *σ*^0^ master; all in dB).

The same amplitude change detection approaches were applied to COSMO-SkyMed data. In this case, the SAR processing and generation of ratio maps were undertaken according to the method described in^35^, using GAMMA SAR and Interferometry Software and starting from Level 1A single-look complex slant (SCS) products.

### Meteorological and climatic data

We referred to NOAA NCDC Climate Data for the year 2018. The original total precipitation (rain and/or melted snow) record is reported during the day in inches and hundredths. For the Bosra flood, data were taken from the time series of the King Hussein weather station (Station ID: GHCND:JOM00040265; 32° 21′ 22.87ʺ N, 36° 15′ 33.05ʺ E) in Jordan. No precipitation data for the period of interest were found for the Sergiopolis flood.

We used Climatic Research Unit (CRU) Time-Series (TS)^42^ for the years 2017–2018 to outline the climatic setting of the regions where Bosra and Sergiopolis are located. Diagrams of monthly precipitation (mm/month) were retrieved at the locations: LAT 32.25°, LON 36.25° and LAT 32.75°, LON 36.75° (Bosra); LAT 34.75°, LON 38.25°, LAT 35.25°, LON 38.75° and LAT 35.75°, LON 39.25° (Sergiopolis).

### Topographic, geological and heritage asset data

Location and name of the monuments of Bosra WHS were sourced from maps and documents published in the UNESCO webpage^12,43^. Information about local geology, position and name of heritage and hydraulic structures based on archaeological findings in Sergiopolis was derived from^13,14^. Figures were georeferenced to the WGS84 datum, and shapefile polygons were digitised in ArcMap v.10.6.1. The topographic setting was based on NASA’s 1 arc-sec SRTM global DEM (30 m ground resolution).

## Supplementary information

Supplementary file1 (DOCX 1905 kb)

## Data Availability

Copernicus Sentinel-1 and Sentinel-2 data are available through the Copernicus Open Access Hub. COSMO-SkyMed data can be accessed through a license to use by ASI. Google Earth images (credits: 2020 Maxar Technologies) can be consulted through Google Earth Pro. Global Summary of the Day data for the year 2018 can be accessed from NOAA NCDC Climate Data Portal (https://www.ncdc.noaa.gov/cdo-web/). Climatic Research Unit (CRU) Time-Series (TS) can be accessed through CEDA Archive (https://archive.ceda.ac.uk/). NASA’s 1 arc-sec SRTM global DEM is distributed by USGS via the LP DAAC Global Data Explorer.

## References

[1] Pavlova I, Makarigakis A, Depret T, Jomelli V (2017). Global overview of the geological hazard exposure and disaster risk awareness at world heritage sites. J. Cult. Herit..

[2] Cigna F, Tapete D, Lee K (2018). Geological hazards in the UNESCO World Heritage sites of the UK: From the global to the local scale perspective. Earth Sci. Rev..

[3] Cazenave A (2014). Anthropogenic global warming threatens world cultural heritage. Environ. Res. Lett..

[4] Rayne L (2017). From above and on the ground: geospatial methods for recording endangered archaeology in the middle East and North Africa. Geosci..

[5] Danti M, Branting S, Penacho S (2017). The American schools of oriental research cultural heritage initiatives: monitoring cultural heritage in Syria and Northern Iraq by geospatial imagery. Geoscience.

[6] Cunliffe, E. *et al. Satellite-based Damage Assessment to Cultural Heritage Sites in Syria Contributors*. (UNITAR/UNOSAT, 2014).

[7] Danti MD (2015). Ground-based observations of cultural heritage incidents in Syria and Iraq. Near East. Archaeol..

[8] Tapete D, Cigna F (2018). Appraisal of opportunities and perspectives for the systematic condition assessment of heritage sites with copernicus Sentinel-2 high-resolution multispectral imagery. Remote Sens..

[9] Tapete D, Cigna F (2019). Detection of archaeological looting from space: methods, achievements and challenges. Remote Sens..

[10] ASOR cultural heritage initiatives. *CHI-April 2018 Report-NEA-PSHSS-14-001 Weekly Report 185–188—April 1–30, 2018.SHI 18-0091*. (2018).

[11] The-day-after-heritage-protection-initiative. *The Day After | Damage Report on Roman Amphitheater in Bosra Al-Sham: May 2, 2018*. (2018).

[12] DGAM. *Ancient city of Bosra. Application document for minor boundary modification*. (2016).

[13] Beckers B, Schütt B (2013). The elaborate floodwater harvesting system of ancient Resafa in Syria: construction and reliability. J. Arid Environ..

[14] Beckers B, Berking J, Schutt B (2012). The elaborated ancient water supply system of Resafa: risk and uncertainty of water harvesting in the Syrian Desert Steppe. ETopoi. J. Anc. Stud..

[15] DGAM. *Bosra WHS Periodic Reporting Cycle 1, Section II / Convention du Patrimoine mondial culturel et naturel Exercice de suivi périodique des sites arabes inscrits sur la liste du Patrimoine mondial*. (2000).

[16] AAAS. Ancient history, modern destruction: assessing the current status of Syria’s world heritage sites using high-resolution satellite imagery | AAAS. The World’s Largest General Scientific Society. (2014). Available at: https://www.aaas.org/page/ancient-history-modern-destruction-assessing-current-status-syria-s-world-heritage-sites-using.

[17] DGAM. *State Party Report on the State of Conservation of the Syrian Cultural Heritage Sites (Syrian Arab Republic)*. (2019).

[18] UNESCO-World-Heritage-Committee. *UNESCO World Heritage Centre: Decision: 37 COM 8C.1*. (2013).

[19] Harris I, Jones PD, Osborn TJ, Lister DH (2014). Updated high-resolution grids of monthly climatic observations: the CRU TS310 Dataset. Int. J. Climatol..

[20] Casana J (2015). Satellite imagery-based analysis of archaeological looting in Syria. Near East. Archaeol..

[21] Danti, M. D., Ali, C., Casana, J. & Prescot, K. W. ASOR Syrian Heritage Initiative (SHI): Planning for Safeguarding Heritage Sites in Syria NEA-PSHSS­14­001 Weekly Report 9: October 6, 2014. (2014). Available at: https://www.asor.org/wp-content/uploads/2019/09/ASOR_CHI_Weekly_Report_09r.pdf.

[22] Danti, M. D., Casana, J., Paulette, T., Franklin, K. & Ali, C. *ASOR cultural heritage initiatives (CHI): planning for safeguarding heritage sites in Syria and Iraq NEA-PSHSS‐14-001 Weekly Report 25: January 26, 2015*. (2015).

[23] Danti, M. D. *et al.* ASOR cultural heritage initiatives (CHI): planning for safeguarding heritage sites in Syria and Iraq NEA—PSHSS-14­001 Weekly Report 28: February 16, 2015. (2015). Available at: https://www.asor.org/wp-content/uploads/2019/09/ASOR_CHI_Weekly_Report_28r.pdf.

[24] Floodlist. Middle East—over 20 dead, hundreds displaced after floods in Syria, Iran and Jordan—FloodList. (2018). Available at: https://floodlist.com/asia/syria-iran-jordan-floods-october-2018.

[25] Brinker W (1991). Zur Wasserversorgung von Resafa-Sergiupolis. Damaszener Mitteilungen.

[26] Agapiou A, Alexakis DD, Hadjimitsis DG (2019). Potential of virtual earth observation constellations in archaeological research. Sensors.

[27] European-Commission. Copernicus in brief | Copernicus. (2015). Available at: https://www.copernicus.eu/en/about-copernicus/copernicus-brief.

[28] European-Commission. Contributing Missions | Copernicus. Available at: https://www.copernicus.eu/en/contributing-missions.

[29] European Space Agency. *Sentinel-1: ESA’s Radar Observatory Mission for GMES Operational Services*. (ESA CommunicationsESTEC, PO Box 299, 2200 AG Noordwijk, The Netherlands, 2012).

[30] Tapete D, Cigna F (2017). Trends and perspectives of space-borne SAR remote sensing for archaeological landscape and cultural heritage applications. J. Archaeol. Sci. Rep..

[31] Sentinel-2-Data products: sentinel handbook. Available at: https://sentinels.copernicus.eu/web/sentinel/missions/sentinel-2/data-products.

[32] Sinergise. Sentinel-2 L2A products available on Sentinel Hub-Sentinel Hub Blog-Medium. (2017). Available at: https://medium.com/sentinel-hub/sentinel-2-l2a-products-available-on-sentinel-hub-beab58903285.

[33] Covello F (2010). COSMO-SkyMed an existing opportunity for observing the Earth. J. Geodyn..

[34] Caltagirone, F. *et al.* The COSMO-SkyMed dual use earth observation program: Development, qualification, and results of the commissioning of the overall constellation. *IEEE J. Sel. Top. Appl. Earth Obs. Remote Sens.* **7**, 2754–2762 (2014).

[35] Tapete D, Cigna F (2019). COSMO-SkyMed SAR for detection and monitoring of archaeological and cultural heritage sites. Remote Sens..

[36] Battagliere ML, Virelli M, Lenti F, Lauretta D, Coletta A (2019). A review of the exploitation of the operational mission COSMO-SkyMed: global trends (2014–2017). Sp. Policy.

[37] Boschetti M, Nutini F, Manfron G, Brivio PA, Nelson A (2014). Comparative analysis of normalised difference spectral indices derived from MODIS for detecting surface water in flooded rice cropping systems. PLoS ONE.

[38] Terradue. SNAC—SNAP S-1 GRD amplitude change—geohazards thematic exploitation platform 2.1 documentation. Available at: https://terradue.github.io/doc-tep-geohazards/tutorials/rss_snap_s1_snac.html.

[39] Foumelis, M. *et al.* Monitoring Geohazards using on-demand and systematic services on Esa’s Geohazards exploitation platform. in *International Geoscience and Remote Sensing Symposium (IGARSS)* 5457–5460 (IEEE, 2019). 10.1109/IGARSS.2019.8898304

[40] Cigna F, Tapete D, Lasaponara R, Masini N (2013). Amplitude change detection with ENVISAT ASAR to image the cultural landscape of the Nasca Region Peru. Archaeol. Prospect..

[41] Terradue. COIN—Coherence and Intensity change for Sentinel-1—Geohazards Thematic Exploitation Platform 2.1 documentation. Available at: https://terradue.github.io/doc-tep-geohazards/tutorials/rss_snap_s1_coin.html.

[42] University of East Anglia Climatic Research Unit, Harris, I.C.; Jones, P. D. Dataset Record: CRU TS4.03: Climatic Research Unit (CRU) Time-Series (TS) version 4.03 of high-resolution gridded data of month-by-month variation in climate (Jan. 1901–Dec. 2018). Available at: https://catalogue.ceda.ac.uk/uuid/10d3e3640f004c578403419aac167d82.

[43] UNESCO. Ancient City of Bosra: Maps-UNESCO World Heritage Centre. Available at: https://whc.unesco.org/en/list/22/multiple=1&unique_number=2209.

