# Supplementary material for: Poorly known 2018 floods in Bosra UNESCO site and Sergiopolis in Syria unveiled from space using Sentinel-1/2 and COSMO-SkyMed
Source: Sci Rep. 2020 Jul 23;10:12307. doi: 10.1038/s41598-020-69181-x (PMC7378183; doi:10.1038/s41598-020-69181-x)
Supplement: Supplementary file 1 — Supplementary file1 (DOCX 1905 kb) [file 41598_2020_69181_MOESM1_ESM.docx]

**Poorly known 2018 floods in Bosra UNESCO site and Sergiopolis in Syria unveiled from space using Sentinel-1/2 and COSMO-SkyMed**

Deodato Tapete ^1,^*, Francesca Cigna ^1^

^1^ Italian Space Agency (ASI), Via del Politecnico snc, 00133 Rome, Italy; francesca.cigna@asi.it

***** Correspondence: deodato.tapete@asi.it

**Supplementary material**

Figure S1 shows the spectral analysis of the pre- to cross-event Sentinel-2 images covering Bosra UNESCO World Heritage Site (WHS) for the 10 control points: 1) Roman Theatre; 2) Birket al-Hajj (or Pilgrims’ Pool); 3-5) the Field (Hippodrome); 6) lot of bare land; 7) East Pool; 8) water reservoir west; 9-10) roads around the theatre. Spectra: (a) prior to the event (23 April 2018), (b) on the day the thunderstorm started (26 April 2018), (c) in the aftermath (3 May 2018). Spectra in (b) show the effect of sky darkness due to nimbus clouds at the beginning of the rainfall event that caused flooding, while spectra in (c) allow for the discrimination between flooded and non-flooded monuments within the WHS.


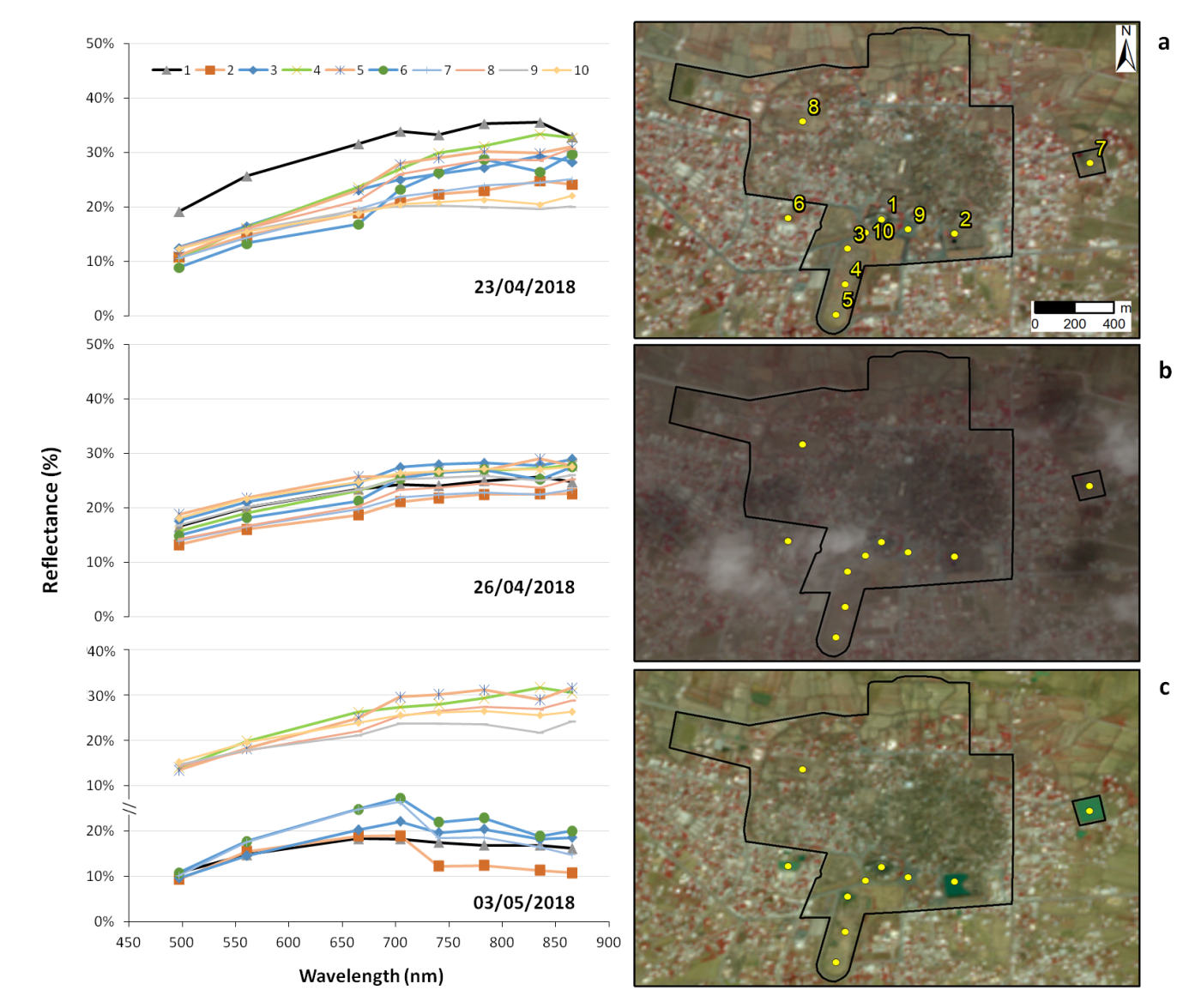


**Supplementary Figure S1.** Spectral analysis of pre- and cross-event Sentinel-2 images over Bosra WHS. Contains Copernicus Sentinel-2 data 2018, displayed as false-coloured IR composites (R: Band 8 – NIR; G: Band 4 – red; B: Band 3 – green; 10 m spatial resolution). Plots were generated by the authors using Microsoft Office Excel 365 (https://www.microsoft.com/en-us/microsoft-365/excel). Maps were generated by the authors using ArcMap v.10.6.1 software (https://desktop.arcgis.com/en/).

On 23 April (pre-event scenario), no signs of flooding were detected and soil conditions were mostly dry (Fig. S1a), with reflectance (%) spanning on average between 10% at Band 2 (492 nm) and 33% at Band 8A (865 nm).

On 26 April (i.e. the day when The Day After Heritage Protection Initiative - TDA stated that the thunderstorm started), an overall decrease of reflectance is observed and the spectra appear flattened (Fig. S1b). This reflects the widespread darkness of the sky due to nimbus clouds above Bosra, visible in the Sentinel-2 image collected early morning at 08:30 UTC.

On 3 May (i.e. the day after the TDA report was issued), the spectra confirm the presence of liquid water (i.e. flooding) within the Roman Theatre, the Birket al-Hajj and the East Pool, as well as in the northern part of the hippodrome (spectra #1-3 and 7 in Fig. S1c). Interestingly, while the water reservoir west was not flooded (#8 in Fig. S1c), other areas in the city were. This is the case of a bare land lot (spectrum #6 in Fig. S1c), 100 m by 50 m wide, located west of the Necropolis of Tell Aswad and the Odeon.

No accurate spectra profiles could be retrieved from the moat at the base of the Ayyubid-era ramparts surrounding the theatre, due to the limited visibility of that part of the monument and the consequent risk of spectral mixing between neighbouring pixels belonging to different objects. On the other side, it is clear that the roads surrounding the theatre and citadel (#9-10 in Fig. S1c) were almost dry.
